# The effect of the ratio of standardized ileal digestible lysine to metabolizable energy on growth performance, blood metabolites and hormones of lactating sows

**DOI:** 10.1186/2049-1891-3-11

**Published:** 2012-03-27

**Authors:** Lingfeng Xue, Xiangshu Piao, Defa Li, Pengfei Li, Rongfei Zhang, Sung Woo Kim, Bing Dong

**Affiliations:** 1 Ministry of Agriculture Feed Industry Centre, State Key Laboratory of Animal Nutrition, China Agricultural University, Beijing 100193, China; 2 Department of Animal Science, North Carolina State University, Raleigh, NC 27695, USA

**Keywords:** Blood metabolites, Hormones, Lactating sows, Metabolizable energy, Performance, Standardized ileal digestible lysine

## Abstract

A total of 335 lactating sows (Landrace × Large White) were used in two experiments to determine the optimum ratio of standardized ileal digestible lysine (SID-Lys) to metabolizable energy (ME) for mixed parity sows during lactation. In Exp. 1, 185 sows (weighing an average of 256.2 ± 6.5 kg and having an average parity of 3.4 ± 0.3) were allocated to one of six experimental diets in a completely randomized block design within parity groups (1, 2, and 3+). The experimental diets were formulated to contain 3.06, 3.16, 3.20, 3.25, 3.30 or 3.40 Mcal/kg of ME and each diet was fed to the sows throughout a 28 day lactation. All diets provided a similar SID-lysine level (0.86%). As a result, the diets provided a SID-Lys:ME ratio of 2.81, 2.72, 2.69, 2.65, 2.61 or 2.53 g/Mcal ME. Sow feed intake was significantly (*P *< 0.01) affected by the energy content of the diet as well as by sow parity. Using regression analysis, feed intake was shown to be maximized at 3.25, 3.21, 3.21 and 3.21 Mcal/kg of ME for parity 1, 2, 3+ sows and the entire cohort of sows respectively (quadratic; *P *< 0.01). In addition, the result of feed intake can be expressed as 2.65, 2.69, 2.69 and 2.68 g/Mcal based on analysis of SID-Lys:ME ratio. Litter weight gain was affected by dietary treatment for parity 3+ sows and the entire cohort (*P *< 0.01). Based on regression analysis, litter weight gain was maximized at 3.25 and 3.24 Mcal/kg of ME for parity 3+ (quadratic; *P *< 0.01) and the entire cohort (quadratic; *P *< 0.01). Similarly, the result of litter weight gain could be expressed as 2.65 and 2.66 g/Mcal of SID-Lys:ME ratio. Therefore, 3.25 Mcal/kg of ME was selected for Exp. 2 in which 150 sows (weighing 254.6 ± 7.3 kg and having an average parity of 3.4 ± 0.4) were allocated to one of five treatments in a completely randomized block design within parity (1, 2, and 3+). The experimental diets were formulated to contain 2.1, 2.4, 2.7, 3.0 or 3.3 g/Mcal of SID-Lys:ME ratio with all diets providing 3.25 Mcal/kg of ME. The diets were fed to the sows throughout a 28 day lactation. Sow body weight loss was affected by dietary treatment (parity 3+ sows, *P *= 0.02; entire cohort, *P *< 0.01) and by sow parity (*P *< 0.01). Litter weight at weaning and litter weight gain were affected by dietary treatment for parity 1, 2, 3+ sows and the entire cohort (*P *< 0.01) as well as by sow parity (*P *< 0.01). Plasma urea nitrogen (*P *< 0.01), creatinine (*P *< 0.01) and non-esterifide fatty acids (*P *= 0.04) were decreased as the SID-Lys:ME ratio of the diet increased. Insulin-like growth factor-1 (*P *= 0.02), estradiol (*P *< 0.01) and luteinizing hormone (*P *= 0.02) were increased as the SID-Lys:ME ratio in diet increased. Based on a broken-line model, the estimated SID-Lys:ME ratio to maximize litter weight gain was estimated to be 3.05 g/Mcal.

## Background

Inadequate nutrient intake of sows during lactation can adversely affect their subsequent reproductive performance [1]. Lysine is considered the first-limiting amino acid in typical corn-soybean meal diets fed to lactating sows [2]. For this reason, more research has focused on identifying the lysine requirements of lactating sows than for any other amino acid.

When formulating diets for lactating sows, it is common practice to provide lysine at a certain percentage of the diet. However, feed intake decreases as the dietary energy concentration increases [3-5] and as a result, total lysine intake may decline as the energy concentration of the diet increases.

Energy restriction has a detrimental effect on sow weight loss throughout lactation, weaning litter weight and weaning-to-estrus interval [6,7]. Thus, an optimum lysine to energy ratio is important to optimize sow reproductive performance. At present, amino acid requirements are often expressed as standardized ileal digestible (SID) values for feed formulation [8]. Therefore, the SID-lysine to ME ratio may be the optimum method of expressing the lysine requirement of lactating sows.

Studies of the optimum lysine to energy ratio have mainly focused on young pigs [9-11]. Studies related to lactating sows are relatively scarce. Therefore, the objective of these experiments was to determine the optimum dietary SID-Lys:ME ratio in the diet of lactating sows under local commercial pig farm conditions.

## Materials and methods

The Animal Welfare Committee of China Agricultural University (Beijing, China) approved the animal care protocol used for this experiment.

### Exp. 1

#### Animals, dietary treatment and management

A total of 185 sows (Landrace × Large White), including 36 first parity, 36 second parity, and 113 third-to-ninth parity sows, were used in this experiment. When the sows exhibited estrus, they were mated twice at 12 h intervals using a mature Duroc boar. After mating, the sows were provided 2 kg/day of a commercially prepared gestation diet providing 3.05 Mcal/kg of ME and 0.65% total lysine from day 0 to 80 of gestation while 3 kg/day was provided from day 80 until day 110.

On day 110 of gestation, all sows were moved into a farrowing room and allocated to one of six corn-soybean meal based diets (Table 1) in a completely randomized block design within parity groups. The experimental diets containing 3.06, 3.16, 3.20, 3.25, 3.30 or 3.40 Mcal/kg of ME were formulated using corn, soybean meal, and wheat bran as their main ingredients. Increased ME concentration was attained by using more corn, soybean meal and soybean oil but less wheat bran. Crystalline L-lysine was added to the diets to ensure that all diets provided a similar SID-lysine level (0.86%). As a result, the diets provided a SID-Lys:ME ratio of 2.81, 2.72, 2.69, 2.65, 2.61 or 2.53 g/Mcal ME. Levels of all other nutrients met or exceeded those recommended by the Agriculture Industry Standard of China [12]. Each treatment had similar numbers of sows in each parity group (1, 2, and 3+, respectively) and the sows were fed the experimental diets as a mash throughout a 28 day lactation.

**Table 1:** Ingredient composition and chemical analysis of diets containing 3.06 to 3.40 Mcal/kg of ME fed to sows in Exp. 1 (as fed basis)

	ME level, Mcal/kg					
	
	3.06	3.16	3.20	3.25	3.30	3.40
Ingredient, %						

Corn	60.00	64.00	64.01	63.01	61.52	64.01

Soybean meal (CP, 43%)	22.81	27.00	27.40	27.50	28.00	28.40

Wheat bran	13.00	5.00	4.00	4.00	4.00	0.00

Soybean oil	--	--	0.60	1.50	2.50	3.60

Limestone	1.64	1.64	1.64	1.64	1.64	1.64

Salt	0.60	0.60	0.60	0.60	0.60	0.60

Dicalcium phosphate	0.84	0.84	0.84	0.84	0.84	0.84

L-Lysine HCl (98%)	0.22	0.12	0.11	0.11	0.10	0.11

Methionine hydroxy analogue^1^	0.04	--	--	--	--	--

L-Threonine (98.5%)	0.05	--	--	--	--	--

Vitamin-mineral premix^2^	0.80	0.80	0.80	0.80	0.80	0.80

Analyzed composition, %						

Crude protein	17.46	17.44	17.48	17.88	17.94	17.89

Lysine	0.97	0.99	0.97	1.01	1.00	0.99

Threonine	0.66	0.67	0.65	0.66	0.68	0.67

Valine	0.80	0.84	0.84	0.83	0.86	0.84

Methionine + cysteine	0.56	0.57	0.57	0.58	0.57	0.56

Tryptophan	0.20	0.21	0.22	0.20	0.22	0.21

Calcium	0.96	0.97	0.97	0.97	0.97	0.97

Phosphorus	0.47	0.47	0.47	0.47	0.47	0.46

Calculated composition						

ME, Mcal/kg^3^	3.06	3.16	3.20	3.25	3.30	3.40

SID-lysine, %^4^	0.86	0.86	0.86	0.86	0.86	0.86

SID-Lys:ME, g/Mcal	2.81	2.72	2.69	2.65	2.61	2.53

^1^DL-methionine hydroxy analogue (84%) provided by Novus International, St. Louis, MO

^2^Premix supplied per kilogram of diet: Vitamin A, 12,000 IU; vitamin D_3_, 2,000 IU; vitamin E, 24 IU; vitamin K_3_, 2.0 mg; vitamin B_1_, 2.0 mg; vitamin B_2_, 6.0 mg; vitamin B_6_, 4.0 mg; vitamin B_12_, 24 μg; niacin, 30 mg; pantothenic acid, 20 mg; folic acid, 3.6 mg; biotin, 0.40 mg; choline, 0.40 g; iron, 96 mg; copper, 8.0 mg; zinc, 120 mg; manganese, 40 mg; iodine, 0.56 mg; selenium, 0.40 mg; and phytase, 120 mg

^3^The metabolizable energy content of corn, soybean meal, and wheat bran were determined previously in our laboratory (data not published). Metabolizable energy of soybean oil was obtained from the Agriculture Industry Standard of China (2004) and was calculated as 96% of DE

^4^Standardized ileal digestible lysine was determined previously using pigs surgically equipped with a T-cannula in the distal ileum. The basal ileal endogenous losses were measured by feeding a nitrogen-free diet (data not published)

During lactation, the sows were housed in individual farrowing crates (2.2 × 1.8 m^2^) equipped with a piglet creep area which was fitted with a heating pad and an infrared heat lamp. Litters were standardized to an average of 10.83 piglets within 48 h of birth by cross-fostering. The experimental diets were provided three times daily at 0630, 1200, and 1700 h. Starting the day after farrowing, the amount of feed provided to the sows was gradually increased until sow maximum intake was reached 7 days postpartum. Any feed remaining in the feed trough was weighed to determine actual daily feed intake. Sows and piglets had free access to water provided by nipple drinkers throughout the experimental period. Piglets were subjected to routine management procedures including teeth clipping, tail docking, ear notching and iron dextran injections (2 mL/pig) within 1 d of parturition. Male piglets were castrated within 7 d after birth and creep feeding was initiated starting 7 days from birth.

#### Measurements

Sows were weighed within 48 h of farrowing and at weaning to assess weight change during lactation. Farrowing date and litter size (number after cross-fostering and at weaning) were recorded. Piglets were weighed and litter weight was recorded after cross-fostering and at weaning.

After weaning at 28 days, sows were moved back to the breeding barn and housed in individual gestation stalls. Detection of estrus was conducted once a day following 15 min of boar stimulation. Estrus was recorded when sows were first observed to show a positive response to the back-pressure test (immobilization reflex) and this value was used to calculate the weaning to estrus interval.

#### Chemical analyses

Analyses for the proximate principles and calcium and phosphorus were carried out according to the Association of Official Analytical Chemists [13]. Amino acids were quantified using an automatic amino acid analyzer (L-8800, Hitachi Incorporated, Tokyo, Japan). Lysine, threonine and valine were analyzed following acid hydrolysis with 6 N HCl for 24 h at 110°C while methionine and cystine were analyzed after cold formic acid oxidation overnight with subsequent hydrolysis. Tryptophan content was determined colorimetrically after alkaline hydrolysis following the procedures described by Miller [14].

### Exp. 2

#### Animals, dietary treatment and management

A total of 150 sows (Landrace × Large White), including 31 first parity, 32 second parity, and 87 third-to-eighth parity sows were used in this experiment. When the sows exhibited estrus, they were mated twice at 12 h intervals using a mature Duroc boar. After mating, the sows were provided 2 kg/day of a commercially prepared gestation diet providing 3.05 Mcal/kg of ME and 0.65% total lysine from day 0 to 80 of gestation while 3 kg/day was provided from day 80 until day 110.

On day 110 of gestation, all sows were moved into a farrowing room and allocated to one of five corn-soybean meal based diets (Table 2) in a completely randomized block design within parity groups. The diets contained SID-Lys:ME ratios of 2.1, 2.4, 2.7, 3.0 or 3.3 g/Mcal. All diets provided 3.25 Mcal/kg ME chosen based on the results of Exp. 1. The requirements for other essential amino acids were met using crystalline amino acids according to the Agriculture Industry Standard of China [12] lactating sow model. Each treatment had a similar number of sows in each parity group (1, 2, and 3+, respectively) and the sows were fed the experimental diets as a mash throughout a 28 day lactation. Sow management, measurements and chemical analyses of diets were conducted as described in Exp. 1.

**Table 2:** Ingredient composition and chemical analysis of diets containing 2.1 to 3.3 g/Mcal of SID-Lys:ME ratio fed to sows in Exp. 2 (as fed basis)

	SID-Lys:ME ratio, g/Mcal				
	**2.1**	**2.4**	**2.7**	**3.0**	**3.3**

Ingredient, %					

Corn	58.70	64.50	64.32	64.06	64.60

Soybean meal, (CP, 43%)	23.00	24.80	24.77	25.00	24.30

Wheat bran	11.56	5.14	4.92	4.47	4.01

Soybean oil	2.80	1.50	1.60	1.70	1.90

Limestone	1.64	1.64	1.64	1.64	1.64

Salt	0.60	0.60	0.60	0.60	0.60

Dicalcium phosphate	0.84	0.84	0.84	0.84	0.84

L-Lysine HCl (98%)	--	0.08	0.21	0.34	0.48

Methionine hydroxy analogue^1^	0.02	0.06	0.14	0.21	0.29

L-Threonine (98%)	--	--	0.03	0.10	0.18

L-Valine (98%)	--	--	0.08	0.17	0.26

L-Tryptophan (98%)	--	--	0.01	0.03	0.06

Vitamin-mineral premix^2^	0.84	0.84	0.84	0.84	0.84

Analyzed composition, %					

Crude protein	17.45	17.63	17.69	17.70	17.67

Lysine	0.80	0.89	1.00	1.08	1.21

Threonine	0.65	0.68	0.70	0.79	0.85

Valine	0.77	0.77	0.86	0.93	1.01

Methionine + cysteine	0.52	0.58	0.66	0.70	0.78

Calcium	0.95	0.95	0.96	0.96	0.94

Phosphorus	0.46	0.47	0.45	0.46	0.47

Calculated composition					

ME, Mcal/kg^3^	3.24	3.25	3.25	3.24	3.24

SID-lysine, %^4^	0.68	0.77	0.87	0.98	1.07

SID-Lys:ME raio, g/Mcal	2.1	2.4	2.7	3.0	3.3

^1^DL-methionine hydroxy analogue (84%) provided by Novus International, St. Louis, MO

^2^Premix supplied per kilogram of diet: Vitamin A, 12,000 IU; vitamin D_3_, 2,000 IU; vitamin E, 24 IU; vitamin K_3_, 2.0 mg; vitamin B_1_, 2.0 mg; vitamin B_2_, 6.0 mg; vitamin B_6_, 4.0 mg; vitamin B_12_, 24 μg; niacin, 30 mg; pantothenic acid, 20 mg; folic acid, 3.6 mg; biotin, 0.40 mg; choline, 0.40 g; iron, 96 mg; copper, 8.0 mg; zinc, 120 mg; manganese, 40 mg; iodine, 0.56 mg; selenium, 0.40 mg; and phytase, 120 mg

^3^The metabolizable energy content of corn, soybean meal, and wheat bran were determined previously in our laboratory (data not published). Metabolizable energy of soybean oil was obtained from the Agriculture Industry Standard of China (2004) and was calculated as 96% of DE

^4^Standardized ileal digestible lysine was determined previously using pigs surgically equipped with a T-cannula in the distal ileum. The basal ileal endogenous losses were measured by feeding a nitrogen-free diet (data not published).

#### Blood sample collection and processing

Blood samples were collected from 10 sows per treatment via jugular vein puncture on day 21 of lactation immediately prior to the morning feeding. Approximately 8 mL of blood was collected for analysis of blood metabolites and hormones. Blood samples were collected in lithium heparin coated vacuum filled tubes (Greiner Bio-One GmbH, Kremsmünster, Australia), placed in an ice box for transportation to the laboratory and centrifuged at 3000 × g for 15 min at 4°C. Plasma samples were stored at -20°C until needed for analysis.

Plasma concentrations of glucose, creatinine, urea nitrogen and non-esterified fatty acids were analyzed using commercial colorimetric kits (BioSino Bio-Technology and Science Incorporated, Bejing, China) and an Automatic Biochemical Analyzer (Hitachi 7160, Hitachi Incorporated, Tokyo, Japan). Plasma insulin, insulin-like growth factor-1, estradiol, luteinizing hormone and follicle-stimulating hormone kits (Beijing Sino-uk Institute of Biological Technology, Beijing, China) were used and their concentrations were measured by radioimmunoassay using an Automatic Radioimmunoassay Counter (R-911, University of Science and Technology of China Industrial Incorporated, Hefei, China).

#### Statistical analysis

Data were analyzed as a randomized complete block design using the General Linear Models (GLM) procedure of SAS V8.2 [15]. Sows were assigned to dietary treatment within parity. There was no treatment x parity interaction (*P *> 0.10) and therefore the interaction effect was removed from the model. Sow body weight at farrowing, lactation length, and weight of the litter after fostering were used as covariates in the analysis. Treatment means were separated using the LSMEANS statement and the PDIFF option in SAS. Linear and quadratic treatment effects were performed using the solution statement in the GLM procedure of SAS. In Exp. 2, a broken-line regression model using the NLMixed procedure of SAS with a random component included for parameter L was used to estimate a break-point according to Robbins et al. [16]. All values are reported as least square means. *P*-values of less than 0.05 were considered statistically significant and *P*-values less than 0.10 were considered as indicative of a significant trend.

## Results

### Exp. 1

#### Sow characteristics

Sow body weight at weaning and body weight change during lactation were affected by parity (*P *< 0.01). First parity sows showed greater body weight loss compared with parity 2 and 3+ sows. Dietary ME level influenced sow body weight loss in parity 1 (*P *= 0.06), 2 (*P *= 0.07) and in the overall cohort of sows (*P *= 0.06). Sow body weight loss decreased quadratically (*P *= 0.01) with increasing levels of ME for the overall cohort of sows. Based on regression analysis, sow body weight loss in the overall cohort of sows was minimized at 3.25 Mcal/kg of ME. Dietary ME level did not affect sow body weight at weaning (Table 3).

**Table 3:** The effect of dietary ME level on sow body weight change in lactation (Exp. 1)

Item	Parity	**No**.	ME level, Mcal/kg								*P*-value		
			
			3.06	3.16	3.20	3.25	3.30	3.40	Mean^13^	SEM	Treat	Linear	Quadratic
Initial sow body weight, kg	1	36	202.6	208.9	205.8	209.4	201.6	214.3	206.8^x^	6.0	0.70	--	--
	
	2	36	235.3	231.9	222.6	224.2	239.0	214.1	227.3^y^	7.7	0.19	--	--
	
	3+	113	273.8	264.1	277.6	274.6	274.2	279.9	274.0^z^	5.8	0.51	--	--
	
	Overall^2^	185	255.9	252.7	256.6	255.9	257.5	258.5	256.2	6.5	0.91	--	--

Sow body weight at weaning, kg	1	36	178.5	185.3	188.4	190.5	181.9	194.3	186.3^x^	6.1	0.48	0.12	0.87
	
	2	36	217.2	211.8	207.7	210.2	223.5	196.0	210.7^y^	7.8	0.19	0.20	0.39
	
	3+	113	258.6	250.2	264.1	260.7	260.0	264.4	259.6^z^	6.0	0.61	0.32	0.73
	
	Overall^2^	185	238.7	236.4	242.3	241.3	242.3	241.9	240.5	6.9	0.83	0.80	0.91

Sow body weight change, kg^1^	1	36	-24.3^a^	-23.5^ac^	-17.5^b^	-18.8^bc^	-20.0^ab^	-19.7^ab^	-19.7^x^	1.9	0.06	0.07	0.08
	
	2	36	-18.0^ab^	-20.0^a^	-14.9^bc^	-14.0^bc^	-15.4^bc^	-18.2^ab^	-16.2^y^	1.6	0.07	0.51	0.07
	
	3+	113	-15.2	-13.6	-13.6	-13.9	-14.2	-15.7	-14.8^y^	1.0	0.65	0.80	0.10
	
	Overall^2^	185	-17.2^a^	-16.3^ab^	-14.3^b^	-14.6^bc^	-15.2^ab^	-16.6^ac^	-15.7	0.9	0.06	0.56	0.01

^1^Initial sow body weight was used as a covariate in the analysis

^2^Parity effect was included in the model

^3^Parity effect; treatment effect was included in the model

^a, b, c^Within a row, values with no common superscript differ significantly (*P *< 0.05)

^x, y, z^Within a column, values with no common superscript differ significantly (*P *< 0.05)

Sow feed intake was affected by parity (Table 4; *P *< 0.01). Higher parity sows consumed more feed during lactation. Dietary ME concentration had a significant effect on feed intake in parity 1 (*P *= 0.07), 2 (*P *< 0.01), 3+ (*P *< 0.01), and the overall cohort of sows (*P *< 0.01). Using regression analysis, it was estimated that feed intake (5.40, 5.77, 5.87, and 5.77 kg for parities 1, 2, 3+, and the overall cohort, respectively) was maximized at 3.25, 3.21, 3.21 and 3.21 Mcal/kg of ME (quadratic, *P *< 0.01). In addition, the result of feed intake can be expressed as 2.65, 2.69, 2.69 and 2.68 g/Mcal based on analysis of SID-Lys:ME ratio,

**Table 4:** The effect of dietary ME level on sow feed intake in lactation (Exp. 1)

Item	Parity	**No**.	ME level, Mcal/kg								*P*-value		
			
			3.06	3.16	3.20	3.25	3.30	3.40	Mean^2^	SEM	Treat	Linear	Quadratic
Feed intake, kg/d	1	36	5.17^a^	5.18^ab^	5.43^bc^	5.46^c^	5.39^abc^	5.21^ab^	5.31^x^	0.10	0.07	0.39	0.01
	
	2	36	5.57^ac^	5.39^ae^	6.00^b^	5.82^bd^	5.65^cd^	5.30^e^	5.63^y^	0.12	< 0.01	0.25	< 0.01
	
	3+	113	5.61^ad^	5.68^a^	5.98^b^	5.92^bc^	5.70^ac^	5.40^d^	5.72^y^	0.09	< 0.01	0.18	< 0.01
	
	Overall^1^	185	5.53^a^	5.54^a^	5.90^b^	5.82^b^	5.64^a^	5.35^c^	5.63	0.07	< 0.01	0.17	< 0.01

Lysine intake, g/d	1	36	50.11^c^	50.22^bc^	53.21^a^	53.55^a^	52.81^ab^	51.07^abc^	51.84^x^	1.01	0.02	0.23	0.01
	
	2	36	54.07^cd^	52.25^d^	58.81^a^	57.00^ab^	55.41^bc^	51.99^d^	54.95^y^	1.25	< 0.01	0.39	< 0.01
	
	3+	113	54.46^cd^	55.08^cd^	58.60^a^	57.99^ab^	55.87^bc^	52.91^d^	55.82^y^	0.94	< 0.01	0.38	< 0.01
	
	Overall^1^	185	52.86^c^	52.98^bc^	56.96^a^	56.28^a^	54.49^b^	51.65^c^	54.20	0.72	< 0.01	0.42	< 0.01

^1^Parity effect was included in the model

^2^Parity effect; treatment effect was included in the model

^a, b, c, d^Within a row, values with no common superscript differ significantly (*P *< 0.05)

^x, y^Within a column, values with no common superscript differ significantly (*P *< 0.05)

#### Litter characteristics

Litter size at weaning and piglet mortality was not influenced by dietary ME level in the overall cohort of sows (Table 5; *P *> 0.10). Dietary ME level did not affect litter weight at weaning and litter weight gain in parity 1 and 2 sows (Table 6; *P *> 0.10). In parity 3+ and the overall cohort of sows, litter weight at weaning and litter weight gain were affected by dietary ME level (*P *< 0.01). Based on regression analysis, litter weight at weaning (80.56 and 78.66 kg) and litter weight gain (2.28 and 2.23 kg/d) were maximized at 3.25 and 3.24 Mcal/kg of ME for parity 3+ (quadratic, *P *< 0.01) and the overall cohort of sows (quadratic, *P *= 0.07, *P *= 0.05, and *P *< 0.01, respectively). Similarly, the result of litter weight gain can be expressed as 2.65 and 2.66 g/Mcal of SID-Lys:ME ratio.

**Table 5:** The effect of dietary ME level on litter size (Exp. 1)

			ME level, Mcal/kg								*P*-value		
**Item**	**Parity**	**No**.	**3.06**	**3.16**	**3.20**	**3.25**	**3.30**	**3.40**	**Mean^13^**	**SEM**	**Treat**	**Linear**	**Quadratic**

No. piglets suckling	1	36	10.80	10.25	10.60	10.40	10.40	10.40	10.49	0.32	0.89	--	--
	
	2	36	10.00	10.40	11.00	11.17	10.83	11.00	10.77	0.34	0.16	--	--
	
	3+	113	10.89	11.26	10.67	10.90	10.95	10.83	10.92	0.25	0.69	--	--
	
	Overall^2^	185	10.73	10.97	10.73	10.88	10.84	10.81	10.83	0.18	0.94	--	--

No. piglets weaned^1^	1	36	9.32	9.94	9.86	9.67	8.88	9.52	9.52	0.40	0.10	0.29	0.76
	
	2	36	9.33^ac^	10.65^b^	9.17^a^	9.88^c^	9.62^ac^	9.84^c^	9.73	0.36	< 0.01	0.09	0.19
	
	3+	113	9.61	9.28	9.91	9.77	9.89	9.46	9.65	0.24	0.24	0.85	0.33
	
	Overall^2^	185	9.52	9.58	9.75	9.76	9.67	9.56	9.64	0.18	0.83	0.67	0.16

Mortality, %^1^	1	36	10.97	5.21	5.75	7.70	15.45	9.30	9.22	2.75	0.10	0.48	0.33
	
	2	36	13.22^ac^	0.65^b^	14.19^a^	8.27^c^	10.77^ac^	8.71^ac^	9.46	2.64	< 0.01	0.82	0.83
	
	3+	113	11.14	14.46	8.95	10.58	9.24	12.97	11.22	2.09	0.28	0.70	0.53
	
	Overall^2^	185	11.44	10.90	9.57	9.81	10.56	11.34	9.52	1.51	0.91	0.93	0.34

^1^Number of piglets suckling and lactation length were used as a covariate in the analysis

^2^Parity effect was included in the model

^3^Parity effect and treatment effect was included in the model

^a, b, c^Within a row, values with no common superscript differ significantly (*P *< 0.05).

**Table 6:** The effect of dietary ME level on litter growth (Exp. 1)

Item	Parity	**No**.	ME level, Mcal/kg								*P*-value		
			
			3.06	3.16	3.20	3.25	3.30	3.40	Mean^13^	SEM	Treat	Linear	Quadratic
Initial litter weight, kg	1	36	13.98	13.35	13.10	13.44	13.92	13.37	13.54^x^	0.35	0.45	--	--
	
	2	36	14.34	14.76	14.84	15.34	15.79	15.47	15.12^y^	0.81	0.86	--	--
	
	3+	113	16.43	16.81	16.51	16.75	16.46	16.25	16.53^z^	0.43	0.95	--	--
	
	Overall^2^	185	15.68	15.94	15.65	15.98	15.96	15.69	15.82	0.38	0.91	--	--

Litter weight at weaning, kg^1^	1	36	72.57	74.63	72.99	73.64	73.35	73.40	73.35^x^	1.89	0.83	0.64	0.18
	
	2	36	76.69	76.65	76.63	76.58	74.93	74.74	75.94^x^	2.25	0.68	0.86	0.71
	
	3+	113	77.33^a^	77.94^ad^	82.66^b^	80.64^c^	79.24^cd^	78.63^ad^	79.39^y^	1.43	< 0.01	0.97	< 0.01
	
	Overall^2^	185	76.42^a^	77.19^a^	80.06^b^	78.83^b^	77.35^a^	76.95^a^	77.80	1.14	< 0.01	0.91	0.07

Litter weight gain, kg/d^1^	1	36	2.08	2.16	2.10	2.12	2.11	2.11	2.11^x^	0.04	0.82	0.67	0.46
	
	2	36	2.17	2.18	2.17	2.17	2.12	2.10	2.15^x^	0.05	0.66	0.56	0.41
	
	3+	113	2.16^a^	2.18^ad^	2.35^b^	2.28^c^	2.23^cd^	2.21^ad^	2.23^y^	0.03	< 0.01	0.29	< 0.01
	
	Overall^2^	185	2.15^a^	2.18^a^	2.28^b^	2.23^b^	2.18^a^	2.17^a^	2.20	0.02	< 0.01	0.69	< 0.01

^1^Initial litter weight, number of piglets suckling and lactation length were used as covariate in the analysis

^2^Parity effect was included in the model

^3^Parity effect; treatment effect was included in the model

^a, b, c, d^Within a row, values with no common superscript differ significantly (*P *< 0.05)

^x, y, z^Within a column, values with no common superscript differ significantly (*P *< 0.05)

#### Subsequent sow performance

The average weaning-to-estrus interval for this experiment was 5.55 ± 0.25 d (Table 7). There was no effect of dietary ME level on the weaning-to-estrus interval for any of the parity groups of sows (*P *> 0.10). Parity 3+ sows had the shortest weaning-to-estrus interval (*P *< 0.01).

**Table 7:** The effect of dietary ME level on the weaning-to-estrus interval of sows^1 ^(Exp. 1)

Item	Parity	**No**.	ME level, Mcal/kg								*P*-value		
			
			3.06	3.16	3.20	3.25	3.30	3.40	Mean^3^	SEM	Treat	Linear	Quadratic
Weaning to estrus interval, d	1	26	6.50	6.50	6.00	6.25	6.20	5.60	6.16^x^	0.54	0.87	0.23	0.72
	
	2	25	6.25	6.00	5.50	5.50	5.60	5.75	5.77^xy^	0.45	0.86	0.35	0.35
	
	3+	79	5.69	5.62	5.15	5.14	5.15	5.31	5.34^y^	0.32	0.72	0.25	0.35
	
	Overall^2^	130	5.93	5.82	5.35	5.39	5.42	5.40	5.55	0.25	0.38	0.09	0.34

^1^Data on 130 sows. Sows which did not exhibit estrus within 20 days were excluded (not induced by the treatment effect). The sows were washed out largely caused by other factors (such as leg problems, mastitis)

^2^Parity effect was included in the model

^3^Parity effect and treatment effect was included in the model

^x, y^Within a column, values with no common superscript differ significantly (*P *< 0.05)

### Exp. 2

#### Sow characteristics

Sow body weight loss was affected by parity (*P *< 0.01). First parity sows had greater body weight loss compared with parity 2 and 3+ sows. Dietary SID-Lys:ME ratio influenced sow body weight loss in parity 3+ (*P *= 0.02) and the overall cohort of sows (*P *< 0.01) and sow body weight loss decreased linearly (*P *< 0.01) with increasing SID-Lys:ME ratio (Table 8).

**Table 8:** The effect of dietary SID-Lys:ME ratio on sow body weight change in lactation (Exp. 2)

Item	Parity	**No**.	SID-Lys:ME ratio, g/Mcal							*P*-value		
			
			2.1	2.4	2.7	3.0	3.3	Mean^13^	SEM	Treat	Linear	Quadratic
Initial sow body weight, kg	1	31	200.2	204.0	199.1	201.8	207.8	202.3^x^	4.27	0.71	--	--
	
	2	32	228.9	220.8	221.2	236.1	227.0	226.6^y^	6.58	0.50	--	--
	
	3+	87	289.4	284.6	286.0	278.5	279.1	283.5^z^	5.57	0.60	--	--
	
	Total	150	256.5	253.6	255.7	252.1	255.0	254.6	7.34	0.99	--	--

Sow body weight at weaning, kg	1	31	178.5	184.7	179.9	182.2	190.3	182.6^x^	4.69	0.50	0.19	0.53
	
	2	32	209.1	203.4	203.8	220.9	211.8	209.7^y^	6.61	0.35	0.32	0.91
	
	3+	87	272.3	268.6	271.9	264.0	264.9	268.3^z^	5.65	0.77	0.30	0.98
	
	Total	150	237.8	236.6	239.8	236.3	240.1	238.1	7.65	0.99	0.84	0.90

Sow body weight change, kg^1^	1	31	-21.8	-19.3	-19.3	-19.7	-17.4	-19.2^x^	1.83	0.66	0.16	0.90
	
	2	32	-19.8	-17.7	-17.6	-14.8	-15.2	-16.8^y^	1.44	0.11	0.01	0.49
	
	3+	87	-17.2^b^	-16.0^ab^	-14.2^ab^	-14.4^a^	-14.1^a^	-15.4^y^	0.80	0.02	< 0.01	0.14
	
	Total^2^	150	-18.5^c^	-16.8^bc^	-15.6^ab^	-15.5^ab^	-14.9^a^	-16.3	0.74	< 0.01	< 0.01	0.23

^1^Initial sow body weight was used as a covariate in the analysis

^2^Parity effect was included in the model

^3^Parity effect and treatment effect was included in the model

^a, b, c^Within a row, values with no common superscript differ significantly (*P *< 0.05)

^x, y, z^Within a column, values with no common superscript differ significantly (*P *< 0.05)

Sow feed intake was affected by parity (Table 9; *P *< 0.01). Higher parity sows consumed more feed during lactation. Dietary lysine intake increased linearly (*P *< 0.01) with increasing SID-Lys:ME ratio as targeted.

**Table 9:** The effect of dietary SID-Lys:ME ratio on sow feed intake in lactation (Exp. 2)

Item	Parity	**No**.	SID-Lys:ME ratio, g/Mcal							*P*-value		
			
			2.1	2.4	2.7	3.0	3.3	Mean^2^	SEM	Treat	Linear	Quadratic
Feed intake, kg/d	1	31	5.40	5.42	5.43	5.36	5.41	5.40^x^	0.12	1.00	0.89	0.98
	
	2	32	5.53	5.51	5.50	5.48	5.49	5.50^x^	0.12	1.00	0.80	0.90
	
	3+	87	5.75	5.62	5.66	5.74	5.78	5.71^y^	0.07	0.42	0.38	0.19
	
	Total^1^	150	5.67	5.60	5.61	5.64	5.67	5.64	0.06	0.80	0.58	0.33

Lysine intake, g/d	1	31	42.68^d^	48.20^c^	54.29^b^	57.86^b^	65.41^a^	53.70^x^	3.32	< 0.01	< 0.01	0.85
	
	2	32	43.67^e^	49.00^d^	54.96^c^	59.20^b^	66.39^a^	54.64^x^	3.42	< 0.01	< 0.01	0.89
	
	3+	87	45.44^e^	50.03^d^	56.62^c^	62.03^b^	69.91^a^	56.81^y^	2.19	< 0.01	< 0.01	0.39
	
	Total^1^	150	44.95^e^	49.88^d^	56.14^c^	60.91^b^	68.54^a^	56.08	1.64	< 0.01	< 0.01	0.62

^1^Parity effect was included in the model

^2^Parity effect and treatment effect was included in the model

^a, b, c, d, e^Within a row, values with no common superscript differ significantly (*P *< 0.05)

^x, y^Within a column, values with no common superscript differ significantly (*P *< 0.05)

#### Litter characteristics

**Figure 1:**
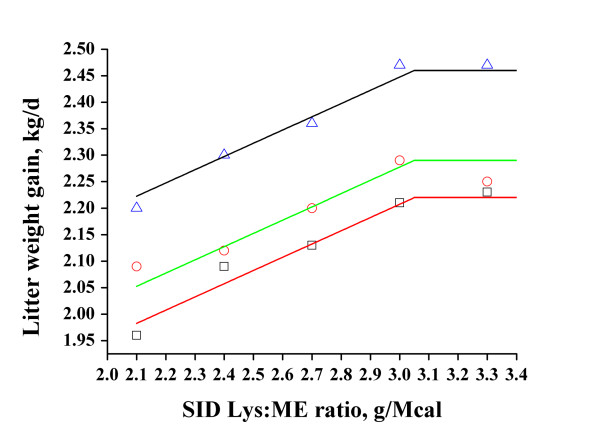
**Fitted broken-line of weight gain as a function of SID-Lys:ME ratio (Exp. 2)**. Observed mean values in each treatment (squares represent parity 1, dots represent parity 2, triangles represent parity 3+) are shown.

Dietary SID-Lys:ME ratio did not influence litter size at weaning and piglet mortality in overall cohort of sows (Table 10; *P *> 0.10). Litter weight at weaning and litter weight gain were affected by dietary SID-Lys:ME ratio for parity 1, 2, 3+ sows and the overall cohort of sows (Table 11; *P *< 0.01). The data were fitted to a straight line regression equation: y = 2.27 - 0.25 × (3.05 - x) + LVAR (R^2 ^= 0.98, adjusted R^2 ^= 0.96). Parameter LVAR represents the parity effect and produced different lines for the respective parities. Based on a broken-line model analysis, the estimated SID-Lys:ME ratio to optimize litter weight gain was 3.05 g/Mcal for mixed parity sows during lactation (Figure 1).

**Table 10:** The effect of dietary SID-Lys:ME ratio on litter size (Exp. 2)

			SID-Lys:ME ratio, g/Mcal							*P*-value		
**Item**	**Parity**	**No**.	**2.1**	**2.4**	**2.7**	**3.0**	**3.3**	**Mean^13^**	**SEM**	**Treat**	**Linear**	**Quadratic**

No. piglets suckling	1	31	10.43	10.67	10.67	10.71	10.60	10.61	0.27	0.95	--	--
	
	2	32	10.83	10.14	10.83	10.83	11.14	10.75	0.39	0.44	--	--
	
	3+	87	11.12	10.94	10.72	10.94	10.78	10.90	0.23	0.78	--	--
	
	Total	150	10.98	10.77	10.78	10.92	10.85	10.86	0.17	0.89	--	--

No. piglets weaned^1^	1	31	9.02	9.17	9.32	8.98	9.42	9.24^x^	0.21	0.62	0.36	0.94
	
	2	32	9.35	9.56	9.27	9.24	9.56	9.42^xy^	0.30	0.77	0.25	0.10
	
	3+	87	9.18^b^	9.52^ab^	9.58^ab^	9.89^a^	9.64^a^	9.53^y^	0.19	0.08	0.14	0.32
	
	Total^2^	150	9.28	9.44	9.45	9.58	9.64	9.48	0.14	0.28	0.03	0.99

Mortality, %^1^	1	31	14.68	13.24	11.83	15.35	10.97	14.17^x^	2.66	0.59	0.84	0.55
	
	2	32	12.41	10.55	13.04	13.96	11.36	12.50^xy^	2.56	0.82	0.62	0.49
	
	3+	87	15.32^b^	11.73^ab^	11.79^ab^	9.19^a^	11.37^a^	11.48^y^	1.78	0.09	0.03	0.12
	
	Total^2^	150	13.56	11.92	12.12	11.31	10.78	11.94	1.29	0.44	0.09	0.51

^1^Number of piglets suckling and lactation length were used as a covariate in the analysis

^2^Parity effect was included in the model

^3^Parity effect and treatment effect was included in the model

^a, b^Within a row, values with no common superscript differ significantly (*P *< 0.05)

^x, y^Within a column, values with no common superscript differ significantly (*P *< 0.05).

**Table 11:** The effect of dietary SID-Lys:ME ratio on the growth of the litter (Exp. 2)

Item	Parity	**No**.	SID-Lys:ME ratio, g/Mcal							*P*-value		
			
			2.1	2.4	2.7	3.0	3.3	Mean^13^	SEM	Treat	Linear	Quadratic
Initial litter weight, kg	1	31	13.57	13.68	14.26	13.41	13.37	13.65^x^	0.53	0.81	--	--
	
	2	32	15.44	16.30	15.17	16.15	15.59	15.74^y^	0.77	0.85	--	--
	
	3+	87	16.59	16.35	16.48	16.13	16.24	16.36^y^	0.45	0.96	--	--
	
	Total	150	15.66	15.80	15.77	15.50	15.61	15.67	0.38	0.98	--	--

Litter weight at weaning, kg^1^	1	31	68.39^d^	71.81^c^	73.01^bc^	75.28^ab^	76.15^ab^	74.40^x^	2.17	< 0.01	< 0.01	0.84
	
	2	32	74.05^d^	75.07^cd^	77.30^bc^	79.99^a^	78.91^ab^	76.66^y^	1.63	< 0.01	< 0.01	0.96
	
	3+	87	77.56^c^	80.31^b^	81.86^b^	85.02^a^	84.92^a^	81.53^z^	1.31	< 0.01	< 0.01	< 0.01
	
	Total^2^	150	75.74^d^	78.33^c^	79.85^b^	82.60^a^	82.34^a^	79.77	1.16	< 0.01	< 0.01	0.06

Litter weight gain, kg/d^1^	1	31	1.96^c^	2.09^b^	2.13^ab^	2.21^a^	2.23^a^	2.11^x^	0.05	< 0.01	< 0.01	0.13
	
	2	32	2.09^c^	2.12^bc^	2.20^ab^	2.29^a^	2.25^a^	2.19^y^	0.04	< 0.01	< 0.01	0.03
	
	3+	87	2.20^c^	2.30^b^	2.36^b^	2.47^a^	2.47^a^	2.36^z^	0.04	< 0.01	< 0.01	0.20
	
	Total^2^	150	2.16^d^	2.25^c^	2.30^b^	2.40^a^	2.40^a^	2.30	0.03	< 0.01	< 0.01	0.08

^1^Initial litter weight, number of piglets suckling and lactation length were used as covariate in the analysis

^2^Parity effect was included in the model

^3^Parity effect and treatment effect was included in the model

^a, b, c, d^Within a row, values with no common superscript differ significantly (*P *< 0.05)

^x, y, z^Within a column, values with no common superscript differ significantly (*P *< 0.05)

#### Subsequent sow performance

The average weaning-to-estrus interval for this experiment was 5.89 ± 0.17 d (Table 12). Dietary SID-Lys:ME ratio affected the weaning-to-estrus interval for parity 1 (*P *< 0.01), 3+ (*P *= 0.06) and the overall cohort of sows (*P *< 0.10). Weaning-to-estrus interval decreased linearly as the SID-Lys:ME ratio of the diets increased (*P *< 0.01). Parity 3+ sows had the shortest weaning-to-estrus interval (*P *< 0.01).

**Table 12:** The effect of dietary SID-Lys:ME ratio on the weaning-to-estrus interval of sows^1 ^(Exp. 2)

Item	Parity	**No**.	SID-Lys:ME ratio, g/Mcal							*P*-value		
			
			2.1	2.4	2.7	3.0	3.3	Mean^3^	SEM	Treat	Linear	Quadratic
Weaning to estrus interval, d	1	24	7.00^b^	6.80^b^	5.75^a^	5.60^a^	5.60^a^	6.16^x^	0.42	< 0.01	< 0.01	0.29
	
	2	26	6.80	6.33	5.80	5.60	5.60	6.03^x^	0.45	0.14	0.01	0.37
	
	3+	79	5.86^c^	5.75^bc^	5.41^abc^	5.19^a^	5.25^ab^	5.50^y^	0.20	0.06	< 0.01	0.51
	
	Total^2^	129	6.44^b^	6.23^b^	5.73^a^	5.51^a^	5.55^a^	5.89	0.17	< 0.01	< 0.01	0.15

^1^Data on 129 sows. Sows which did not exhibit estrus within 20 days were excluded (not induced by the treatment effect). The sows were washed out largely caused by other factors (such as leg problems, mastitis)

^2^Parity effect was included in the model

^3^Parity effect and treatment effect was included in the model

^a, b, c^Within a row, values with no common superscript differ significantly (*P *< 0.05)

^x, y^Within a column, values with no common superscript differ significantly (*P *< 0.05)

#### Plasma characteristics

Dietary treatment did not affect plasma glucose levels. Plasma urea nitrogen (*P *< 0.01), creatinine (*P *< 0.01) and non-esterifide fatty acids (*P *= 0.04) decreased as the SID-Lys:ME ratio of the diets increased and a linear response was observed (Table 13; *P *< 0.01). The dietary SID-Lys:ME ratio influenced plasma insulin-like growth factor-1 (*P *= 0.02), estradiol (*P *< 0.01) and luteinizing hormone (*P *= 0.02) content and these indices increased linearly as the SID-Lys:ME ratio in the diet increased (Table 14; *P *< 0.01).

**Table 13:** The effect of dietary SID-Lys:ME ratio on plasma metabolites of mixed parity sows (Exp. 2)

Item	SID-Lys:ME ratio, g/Mcal						*P*-value		
	
	2.1	2.4	2.7	3.0	3.3	SEM	Treat	Linear	Quadratic
Glucose, mmol/L	5.74	5.78	5.30	5.33	5.41	0.26	0.55	0.18	0.55

Plasma urea nitrogen, mmol/L	5.94^b^	5.92^b^	5.79^b^	4.92^a^	4.80^a^	0.27	< 0.01	< 0.01	0.28

Creatinine, μmol/L	122.3^b^	120.1^b^	118.8^b^	107.6^a^	109.8^a^	3.19	< 0.01	< 0.01	0.92

Non-esterifide fatty acids, mmol/L	0.53^b^	0.53^b^	0.50^ab^	0.48^a^	0.48^a^	0.02	0.04	< 0.01	0.95

^a, b^Within a row, values with no common superscript differ significantly (*P *< 0.05)

**Table 14:** The effect of dietary SID-Lys:ME ratio on plasma metabolic hormones of mixed parity sows (Exp. 2)

Item	SID-Lys:ME ratio, g/Mcal						*P*-value		
	
	2.1	2.4	2.7	3.0	3.3	SEM	Treat	Linear	Quadratic
Insulin, μIU/mL	18.55	18.42	18.68	19.49	18.93	0.47	0.55	0.23	0.86

Insulin-like growth factor-1, ng/mL	185.4^b^	185.0^b^	192.5^ab^	200.4^a^	198.4^a^	4.32	0.02	< 0.01	0.86

Estradiol, pg/mL	23.69^b^	24.44^b^	27.91^a^	28.18^a^	28.29^a^	1.17	< 0.01	< 0.01	0.25

Luteinizing hormone, mIU/mL	6.11^b^	6.13^b^	6.46^ab^	7.52^a^	7.42^a^	0.41	0.02	< 0.01	0.74

Follicle-stimulating hormone, mIU/mL	10.69	10.61	11.29	11.21	11.23	0.51	0.81	0.30	0.78

^a, b^Within a row, values with no common superscript differ significantly (*P *< 0.05)

## Discussion

In the present experiment, it would appear that sows were able to breakdown body tissues to meet the nutrient needs for lactation even when their dietary energy intake was inadequate. The relationship of sow weight loss to dietary energy intake observed during lactation in Exp. 1 agrees with previous reports [17,18]. This suggests that increasing dietary energy concentration could be an important factor to alleviate sow body weight loss during lactation. First parity sows lost more body weight compared with multiparous sows. Primiparous sows are physiologically younger and undergo an intense lactation period which may result in large body tissue catabolism.

Sow body weight loss was minimized at 3.25 Mcal/kg of ME for the overall sow cohort used in Exp. 2. At this energy level, the body weight losses observed in sows during lactation were related to a reduced SID-Lys:ME ratio suggesting that diets with a low SID-Lys:ME ratio may not meet the sow's nutrient requirements for maintenance and milk production. Previous studies reported that an increased lysine intake can minimize body weight loss of lactating sows [19-21]. Sows did not mobilize body tissue as their nutrient intake increased and a lysine intake of 74 g/d did not affect sow body weight change [22].

An increase in feed intake of sows during lactation has been shown, at least partly, to reduce body weight and backfat loss and thereby alleviate the negative impacts of nursing a large litter [23]. However, in Exp. 1, feeding a high dietary energy concentration (3.40 Mcal/kg of ME) reduced feed intake, a finding which is in agreement with previous reports [5,24]. Pettigrew and Moser [25] summarized 19 experiments and found that feed intake was reduced in 16 of the studies in which sows were fed high energy diets. Feed intake was also reduced for the sows fed the lowest ME level, in which the diet was high in wheat bran (3.06 Mcal/kg of ME). In Exp. 2, dietary energy intake was similar to the optimum ME level obtained from Exp. 1 and lysine intake in the groups differed as targeted. The responses to the different SID-Lys:ME ratio diets obtained are mainly a function of the lysine intake of lactating sows.

Litter growth rate was improved for sows fed a higher energy diet during lactation [5]. In Exp. 1, litter performance was influenced as dietary ME level changed for parity 3+ and the overall cohort of sows. However, a ME level of 3.30 or 3.40 Mcal/kg did not improve litter performance. Pluske et al. [26] also reported that when metabolizable energy intake increased from 75 to 104 MJ/d (17.93 to 24.86 Mcal/d) there was no additional weight gain of the litter. At a ME content of 3.25 Mcal/kg obtained from Exp. 1, the litter weight gain increased as the SID-Lys:ME ratio increased in Exp. 2 and the estimated SID-Lys:ME ratio to optimize litter weight gain was 3.05 g/Mcal in mixed parity lactating sows. Other studies reported that litter weight gain was improved by higher lysine intake as well [20,27,28].

Weaning-to-estrus interval is an important factor affecting the overall reproductive efficiency of sows. A short interval is necessary to maximize the number of pigs marketed per sow per year [6]. A high energy intake of sows resulted in a short weaning-to-estrus interval in previous studies [17,18]. Higher-parity sows demonstrated a shorter weaning-to-estrus interval in Exp.1 and 2. The weaning-to-estrus interval was shortened as the SID-Lys:ME ratio increased in Exp.2. Other studies have reported that increasing lysine intake did not affect weaning-to-estrus interval of sows [21,29,30]. These results may be due to the large lactation weight loss for sows rather than the low lysine intake. Vesseur et al. [31] found that sows losing more than 7.5% of their body weight during lactation exhibited a prolonged weaning-to-estrus interval. Similar results were reported by Koketsu et al. [32]. Thaker and Bilkei [33] evaluated data from 1677 sows and observed that the subsequent reproduction performance of sows decreased as weight loss increased during lactation.

Plasma urea nitrogen can represent a sensitive response criterion for protein utilization [34] and its concentrations decreased quadratically with increasing dietary lysine intake [35]. Similar results were found in Exp. 2. These results indicate that sows require a higher dietary SID-Lys:ME ratio to minimize plasma urea nitrogen concentrations and presumably to minimize body protein mobilization.

Plasma creatinine is an indicator of muscle catabolism [28]. Its concentrations decreased when the dietary SID-Lys:ME ratio increased. This is likely a reflection of the fact that sows fed higher lysine lost less weight in lactation and therefore were presumably catabolizing less muscle tissue. Sows body weight loss was positively correlated with non-esterified fatty acid concentrations and their concentration has commonly been used as a postpartum measure of fat catabolism [21]. The lower concentrations could be a sign of a low capacity of the sows to catabolize body fat tissue thus having lower body weight loss [36]. Both implications are verified by the good body condition of sows in Exp. 2.

Plasma insulin-like growth factor-1 level plays an important role in determining nutritional status [37]. Insulin-like growth factor-1 concentrations increased as the lysine intake of lactating sows increased [21,29]. Therefore, the increased concentrations of plasma insulin-like growth factor-1 observed in Exp. 2 suggest an improved metabolic state with a high SID-Lys:ME ratio.

Estradiol is secreted mainly from the ovarian follicles [29]. Sows fed higher SID-Lys:ME ratio diets had higher plasma estradiol concentrations implying that lysine intake could modulate follicular development during lactation. Similar results were found by Yang et al. [29] where the authors found that a high lysine intake increased serum estradiol concentrations during late lactation. Furthermore, a high lysine intake enhanced the proportion of large follicles during proestrus [38]. In Exp. 2, it can be concluded that increasing plasma estradiol may provide a positive effect on ovarian function during late lactation. The shorter weaning-to-estrus interval observed supports this hypothesis.

Luteinizing hormone plays an important role in the reproductive performance of sows. Tokach et al. [39] and Shaw and Foxcroft [40] reported that increased plasma luteinizing hormone concentrations shortened the weaning-to-estrus interval in sows. The shorted duration to return to estrus after weaning was presumed to be associated with an increased secretion of luteinizing hormone. However, inadequate lysine intake might decrease the release of luteinizing hormone during lactation [28,29]. Our data showed that a high SID-Lys:ME ratio in the diet increased luteinizing hormone concentration on d 21 of lactation which might explain the shorter weaning-to-estrus interval.

In conclusion, feeding lactating sows a diet with an optimal ME concentration improved body condition and voluntary feed intake of sows and increased litter growth. Litter growth rate was maximized when ME was 3.25 and 3.24 Mcal/kg for parity 3+ sows and the overall cohort of sows, respectively. This result can be expressed as 2.65 and 2.66 g/Mcal of SID-Lys:ME ratio. Parity had significant effects on body weight loss, voluntary feed intake, and weaning-to-estrus interval of sows. Based on the results obtained in the present studies, the optimum SID-Lys:ME ratio appears to be 3.05 g/Mcal for lactating sows fed at the ME level of 3.25 Mcal/kg in the diets.

## Competing interests

The authors declare that they have no competing interests.

## Authors' contributions

LX carried out the experiments, and performed the statistical analysis and drafted the manuscript. XP and DL conceived of the study, and participated in its design and coordination. PL and RZ participated in the design of the study. SK and BD modified the language manuscript. All authors read and approved the final manuscript.
